# CAMK2N1 inhibits prostate cancer progression through androgen receptor-dependent signaling

**DOI:** 10.18632/oncotarget.2511

**Published:** 2014-09-25

**Authors:** Tao Wang, Shuiming Guo, Zhuo Liu, Licheng Wu, Mingchao Li, Jun Yang, Ruibao Chen, Xiaming Liu, Hua Xu, Shaoxin Cai, Hui Chen, Weiyong Li, Shaohua Xu, Liang Wang, Zhiquan Hu, Qianyuan Zhuang, Liping Wang, Kongming Wu, Jihong Liu, Zhangqun Ye, Jun-Yuan Ji, Chenguang Wang, Ke Chen

**Affiliations:** ^1^ Department of Urology, Tongji Hospital, Tongji Medical College, Huazhong University of Science and Technology, Wuhan, Hubei, China; ^2^ Institute of Urology, Tongji Hospital, Tongji Medical College, Huazhong University of Science and Technology, Wuhan, Hubei, China; ^3^ Department of Surgery, Tongji Hospital, Tongji Medical College, Huazhong University of Science and Technology, Wuhan, Hubei, China; ^4^ Department of Radiology, Tongji Hospital, Tongji Medical College, Huazhong University of Science and Technology, Wuhan, Hubei, China; ^5^ Department of Oncology, Tongji Hospital, Tongji Medical College, Huazhong University of Science and Technology, Wuhan, Hubei, China; ^6^ Union Hospital, Tongji Medical College, Huazhong University of Science and Technology, Wuhan, Hubei, China; ^7^ Kimmel Cancer Center, Department of Cancer Biology, Thomas Jefferson University, Philadelphia, PA, USA; ^8^ Department of Molecular and Cellular Medicine, College of Medicine, Texas A&M University Health Science Center, College Station, TX, USA; ^9^ Department of Gynecology, Shanghai First Matenity and Infant Hospital, Tongji University School of Medicine, Shanghai, China; ^10^ Key Laboratory of Tianjin Radiation and Molecular Nuclear Medicine; Institute of Radiation Medicine, Peking Union Medical College & Chinese Academy of Medical Sciences, Tianjin, China

**Keywords:** CAMK2N1, Androgen receptor (AR), prostate cancer, tumor suppressor

## Abstract

Castration resistance is a major obstacle to hormonal therapy for prostate cancer patients. Although androgen independence of prostate cancer growth is a known contributing factor to endocrine resistance, the mechanism of androgen receptor deregulation in endocrine resistance is still poorly understood. Herein, the CAMK2N1 was shown to contribute to the human prostate cancer cell growth and survival through AR-dependent signaling. Reduced expression of CAMK2N1 was correlated to recurrence-free survival of prostate cancer patients with high levels of AR expression in their tumor. CAMK2N1 and AR signaling form an auto-regulatory negative feedback loop: CAMK2N1 expression was down-regulated by AR activation; while CAMK2N1 inhibited AR expression and transactivation through CAMKII and AKT pathways. Knockdown of CAMK2N1 in prostate cancer cells alleviated Casodex inhibition of cell growth, while re-expression of CAMK2N1 in castration-resistant cells sensitized the cells to Casodex treatment. Taken together, our findings suggest that CAMK2N1 plays a tumor suppressive role and serves as a crucial determinant of the resistance of prostate cancer to endocrine therapies.

## INTRODUCTION

Prostate cancer is one of the most common malignancies in men. As the average life expectancy has been prolonged, the incidence and mortality rates of prostate cancer have increased significantly in recent years [1]. The progression of prostate cancer normally goes from castration-sensitive to castration-resistant, inevitably developing highly metastatic properties [2]. Prostate tumors initially respond to hormonal intervention therapies, however, as androgen-independence emerges, tumors develop resistance [3]. The main treatments for advanced prostate cancer consist of hormone therapy, chemotherapy and/or radiation. Unfortunately, although the diagnosis of cancer has advanced, limited therapy options stall are stalling the survival rates in patients [4, 5].

The CAMK2N1 gene, cloned and characterized as an inhibitor of CaMKII (calcium/calmodulin-dependent protein kinase II), has been shown to affect tumorigenesis and tumor growth [6, 7]. In a prior study, we demonstrated that CAMK2N1 expression was reduced in prostate cancer, and re-introduction of CAMK2N1 significantly impaired human prostate cancer cell proliferation and tumor growth *in vivo* [8]. Genome-wide gene profiling revealed that CAMK2N1 regulated the expression of key genes associated with cell-cycle progression and apoptosis [8]. Furthermore, CAMK2N1 suppressed androgen receptor (AR) mRNA levels and AR regulators such as IGF-1, ErbB2, AKT, and HSP27 [8]. This data suggests that CAMK2N1 plays an important role in the progression of prostate cancer. However, the molecular mechanisms and functional link between CAMK2N1 and AR signaling is still unknown.

In this study, we observed CAMK2N1 and AR signaling form an auto-regulatory negative feedback loop in human prostate cancer cells. CAMK2N1 expression was inversely correlated with AR levels in prostate cancer, and patients with higher CAMK2N1 expression in their tumor have improved recurrence-free survival. Importantly, ectopic expression of CAMK2N1 in castration-resistant prostate cancer cells sensitized cells to response to anti-androgen treatment. Taken together, our findings revealed a tumor suppressive role for CAMK2N1 and established CAMK2N1 as molecular determinant in hormone sensitivity of prostate cancer.

## RESULTS

### A significant negative correlation between CAM2KN1 and AR in clinical prostate cancer specimens

**Figure 1:**
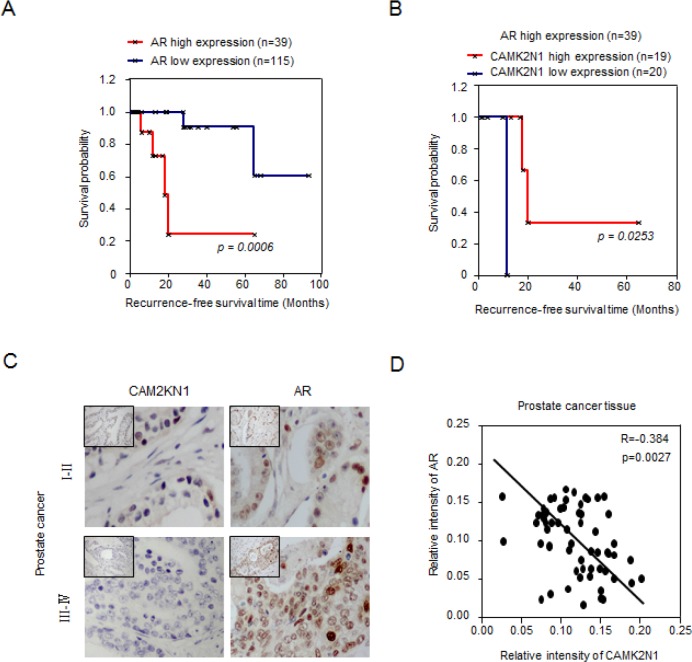
A significant negative correlation between CAM2KN1 and AR in prostate cancer specimens (A) In Kaplan-Meier analysis, patients with tumors expressing high levels of AR (N = 39) had poor recurrence-free survival (*p = 0.0006*). (B) In Kaplan-Meier analysis, there was a significant trend toward improved survival in cases showing high expression of CAMK2N1 (N = 19) as opposed to cases showing low expression of CAMK2N1 (N = 20) in recurrent patients with high AR expression (*p = 0.023*). (C) IHC was also conducted to determine CAMK2N1 and AR expression in the prostate cancer specimens (N = 70). AR and CAMK2N1 were inversely correlated in human prostate cancers (*r* = −0.384, *p* = 0.0027).

The androgen receptor (AR) is a ligand-activated transcription factor, which plays critical roles in normal prostate development and prostate tumorigenesis [3]. The growth of advanced prostate cancer (both castration-sensitive prostate cancer (CSPC) and castration-resistant prostate cancer (CRPC)) depends on androgen receptor signaling. In our prior study, CAMK2N1 suppressed the expression of AR (mRNA levels) and AR regulators including IGF-1, ErbB2, JAK and HSP27 [8]. However, the clinical significance of these observations has not been investigated. We first performed *in silico* analysis for AR and CAMK2N1 expression on a clinical gene-expression array dataset composed of 154 patient samples with follow-up information (Supplemental Figure 1A) [9]. The Kaplan-Meier analysis was conducted to evaluate the difference in recurrence-free survival associated with high versus low expression of AR and CAMK2N1 gene. Genes that correspond to the AR and CAMK2N1 signature were used to assign the samples as high (upper 25th percentile) or low (lower 75th percentile) [10]. In Kaplan-Meier analysis for AR, patients with tumors expressing high AR (n = 39) had significantly lower recurrence-free survival (*p = 0.0006*) (Figure. 1A). In recurrent patients with high AR expression (*p = 0.023*), there was a significant trend toward improved survival of those patients with high expression of CAMK2N1 (n = 19) compared to those with low CAMK2N1 expression (n = 20) in tumors (Figure. 1B). As revealed by IHC to determine the expression of AR and CAMK2N1 in the prostate cancer specimens (n=70) [8]. AR and CAMK2N1 were inversely correlated in human prostate cancer (*r = −0.384, p = 0.0027*) (Figure. 1C-D). Taken together, these results suggested a functional interaction between CAMK2N1 and AR during prostate cancer progression.

### CAMK2N1 expression was down-regulated by androgen/AR signaling

**Figure 2:**
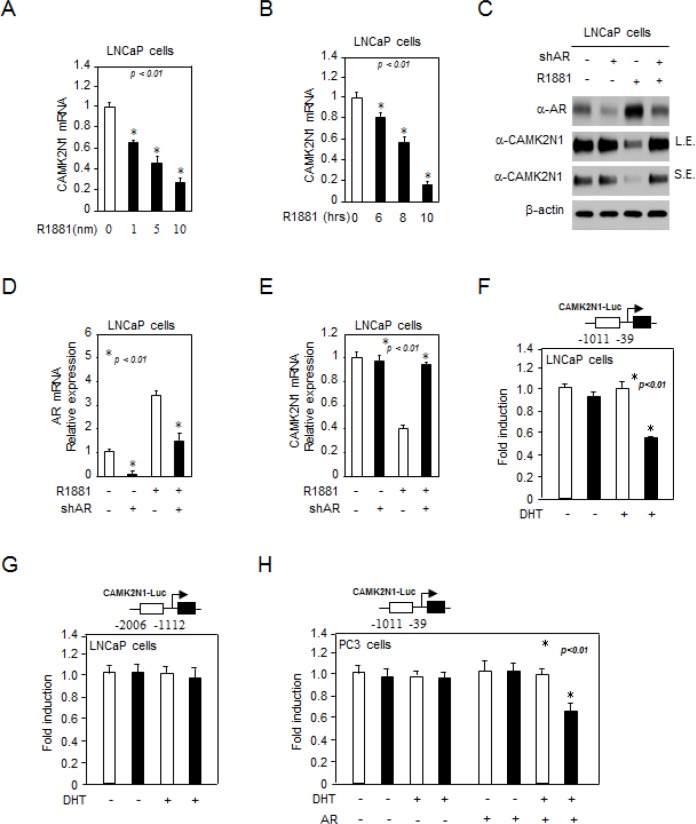
CAMK2N1 expression was down-regulated by androgen/AR signaling (A-B) LNCaP cells were treated with 1-100 nM R1881 for 6-10 hrs. CAMK2N1 mRNA levels were determined by qRT-PCR. (C-E) CAMK2N1, AR protein and mRNA levels were determined by Western blot and qRT-PCR in LNCaP cells with stably knockdown of AR. LNCaP cells were treated with 10 nM R1881 for 10 hrs. (F-H) CAMK2N1 promoter reporters (−39~−1011, −1112~−2006) were assessed in AR-positive LNCaP cells and AR-negative PC3 cells. R1881 repressed activity of CAMK2N1 gene reporters (−39~−1011) in the presence of AR.

The inverse correlation between AR and CAMK2N1 raised a possibility that CAMK2N1 may functionally interact with androgen/AR signaling. To explore this possibility, we first determined whether androgen signaling affects CAMK2N1 expression. Castration-sensitive prostate cancer cells LNCaP and castration-resistant prostate cancer cells C4-2 were treated with 1-10 nM of R1881 for 6-10 hrs and the total RNA was isolated. As shown in Fig. 2A-B, Supplemental Figure 2A, qRT-PCR analysis revealed that CAMK2N1 mRNA levels were decreased upon androgen treatment in LNCaP cells. In addition, we searched the microarray data in the public domains for effects of androgens on CAMK2N1 mRNA expression in AR-positive prostate cancer cell lines [11, 12], and similar observation was also made in this database (Supplemental Figure 2B). Next, our western blot and qRT-PCR analysis showed that CAMK2N1 protein and mRNA levels were decreased in cells treated with androgen, and androgen inhibition of CAMK2N1 was alleviated by shRNA-mediated AR knockdown (Fig. 2C-E). Collectively, these observations indicate that CAMK2N1 is an androgen-responsive gene, which is down regulated by AR activation.

We further performed luciferase reporter assays to evaluate the effect of androgen/AR signaling on CAMK2N1 transactivation. The CAMK2N1 promoter reporters (−39~−1011, −1112~−2006) were assessed in AR-positive LNCaP cells and AR-negative PC3 cells. R1881 repressed activity of CAMK2N1 gene reporters (−39~−1011, but not −1112~−2006) in the presence of AR (Fig. 2F-H). Taken together, these findings indicated that CAMK2N1 expression and transactivation were down-regulated by androgen/AR signaling.

### CAMK2N1 inhibited AR expression and transactivation

**Figure 3:**
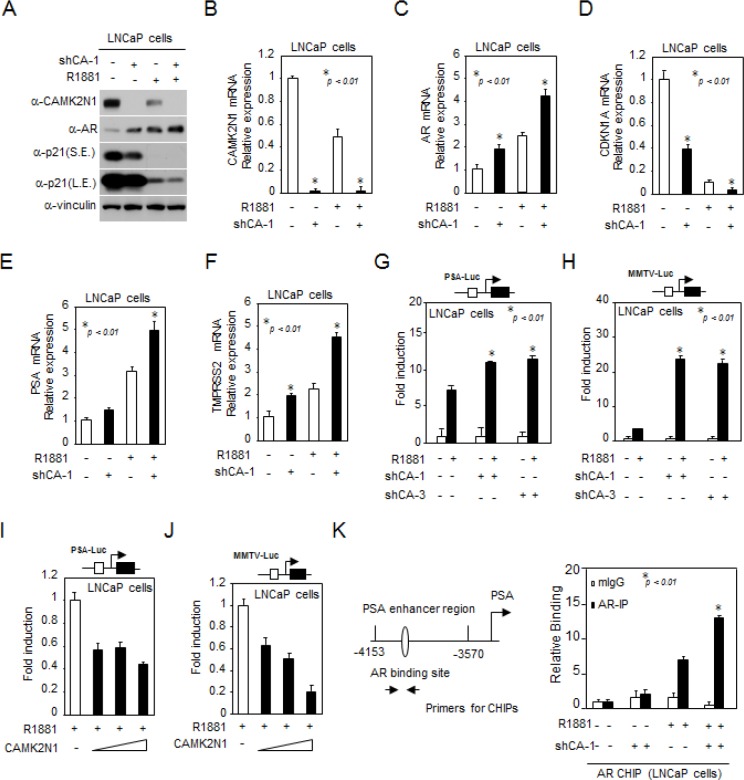
CAMK2N1 inhibited AR expression and transactivation (A) CAMK2N1, AR and p21 protein levels were determined by Western blot in LNCaP cells with stably knockdown of CAMK2N1. LNCaP cells were treated with 10 nM R1881 for 10 hrs. (B-F) CAMK2N1, AR, PSA, TMPRSS2 and p21 mRNA levels were determined by qRT-PCR in LNCaP cells with stably knockdown of CAMK2N1. LNCaP cells were treated with 10 nM R1881 for 10 hrs. (G-J) Androgen-responsive luciferase reporter genes (PSA-Luc, MMTV-Luc) were assessed for AR activity. LNCaP cells with CAMK2N1 overexpression or knockdown were treated with R1881 for 24 hrs. (K) CHIP analysis of AR for PSA promoter region in LNCaP cells with stable knockdown of CAMK2N1. LNCaP cells were treated with R1881 or vehicle for 10 hrs. CHIP assay was performed using an anti-AR antibody.

In order to determine whether CAMK2N1 regulates AR signaling, we conducted western blot and qRT-PCR analysis in LNCaP cells with stable knockdown of CAMK2N1. As shown in Fig. 3A, cells treated with AR agonist R1881 increased AR protein expression and decreased p21 expression. Depletion of CAMK2N1 further enhanced AR expression levels in the presence and absence of the ligand. As shown in Fig. 3B-F, cells treated with AR agonist R1881 increased AR, AR target genes such as PSA and TMPRSS2 mRNA levels, and decreased p21 mRNA levels, and depletion of CAMK2N1 further enhanced AR, PSA and TMPRSS2 mRNA levels in the presence and absence of the ligand.

Furthermore, we conducted luciferase reporter assays to evaluate the effect of CAMK2N1 on AR transactivation. The androgen-responsive promoter reporters were assessed in AR-positive LNCaP cells where R1881 induced activity of androgen responsive gene reporters including PSA-Luc and MMTV-Luc (Fig. 3G-J). Knockdown of CAMK2N1 increased R1881-induced PSA-Luc activity by 2-fold (Fig. 3G) and MMTV-Luc by 24-fold (Fig. 3H). Conversely, Overexpression of CAMK2N1 reduced R1881-induced reporter activity by 50% (Fig. 3I-J). To examine the interaction between AR and CAMK2N1 in the context of local chromatin, we conducted chromatin immunoprecipitation (ChIP) assays. AR has been previously shown to bind to the enhancer elements of the PSA gene (−4153/−3570 bp) [13, 14]. Thus we performed ChIP analysis using anti-AR antibody to pull down PSA enhancer elements. As shown in Fig. 3K, shRNA-mediated knockdown of endogenous CAMK2N1 increased AR binding to the PSA enhancer elements in an androgen-dependent manner. Taken together, these observations suggested that CAMK2N1 inhibits AR expression and AR-dependent transactivation.

### CAMK2N1-mediated suppression of AR transactivation is dependent on the CAMKII and AKT pathways

**Figure 4:**
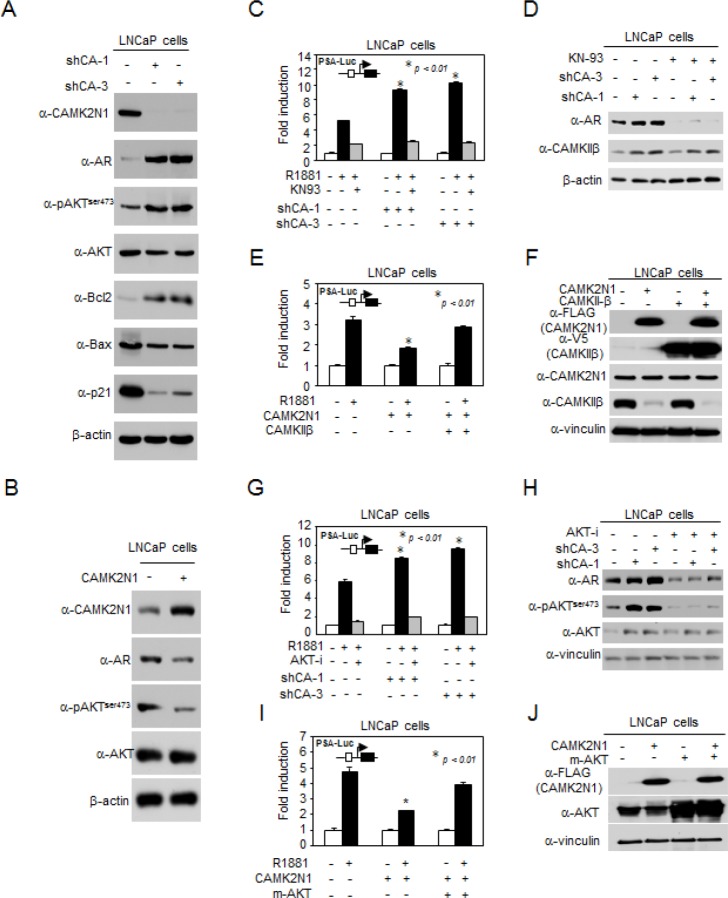
CAMK2N1-mediated suppression of AR transactivation is dependent on the CAMKII and AKT pathways (A) Expression levels of pAKT^ser473^, AKT, AR, Bcl-2, BAX, and p21 were determined by Western blot in LNCaP cells with stable CAMK2N1 knockdown. (B) Expression levels of pAKT^ser473^, AKT and AR were determined by Western blot in LNCaP cells with stable CAMK2N1 overexpression. (C-D) CAMK2N1 knockdown cells treated with 10 nM R1881 and/or 20μM KN-93 inhibitor. PSA-Luc was assessed for AR activity. CAMKIIβ and AR protein levels were determined by Western blot. (E-F) LNCaP cells transiently transfected with CAMK2N1 and/or CAMKIIβ while treated with 10 nM R1881. PSA-Luc was assessed for AR activity. CAMK2N1 and CAMKIIβ protein levels were determined by Western blot. (G-H) CAMK2N1 knockdown cells treated with 10 nM R1881 and/or 20 μM AKT VIII. PSA-Luc was assessed for AR activity. pAKT^ser473^, AKT and AR β protein levels were determined by Western blot. (I-J) LNCaP cells transiently transfected with CAMK2N1 and/or m-AKT. PSA-Luc was assessed for AR activity. CAMK2N1 and CAMKIIβ protein levels were determined by Western blot.

We had previously shown that CAMK2N1 regulated cell proliferation and cell death signaling [8]. To validate CAMK2N1-regulated signaling pathways in CSPC cells, a subset of genes that were AR, AKT, pAKTser473, Bcl-2, p21 and Bax were determined by Western blot. Depletion of CAMK2N1 by shRNA in LNCaP cells resulted in increased AR, pAKTser473, and Bcl-2 expression and decreased p21 and Bax expression (Fig. 4A). Conversely, overexpression of CAMK2N1 in LNCaP cells led to an opposite effect on protein expression of these genes (Fig. 4B).

CAMKII is a ubiquitous serine/threonine protein kinase that phosphorylates nearly 40 different proteins, including enzymes, ion channels, kinases and transcription factors [6, 7]. Previous study demonstrated that CAMKII regulates the PI3K/AKT pathway by phosphorylating AKT at Ser473 [15]. Inhibition of AKT phosphorylation and activity suppresses AR levels in prostate cancer [16]. The current study demonstrated that CAMK2N1, the inhibitor of CAMKII, inhibited AKT phosphorylation at Ser473 and repressed AR expression and activity. In order to further explore the functional interaction between CAMK2N1 and AR, we conducted luciferase reporter and Western blot to determine whether CAMK2N1-mediated suppression of AR activity is dependent on CAMKII and AKT signaling. As shown in Fig. 4C-D, Fig. 4G-H and Supplemental Figure 3G, knockdown of CAMK2N1 increased R1881-induced PSA-Luc activity, whereas addition of CAMKII inhibitor (KN-93) or AKT inhibitor VIII abrogated the effects of CAMK2N1 knockdown on AR activity. Conversely, overexpression of CAMK2N1 decreased R1881-induced PSA-Luc activity, while transient transfection of LNCaP cells with constitutively active CAMKIIβ or AKT (myrAKT) resulted in increased PSA-Luc activity (Fig. 4E-F and Fig. 4I-J). This data indicates that CAMK2N1-mediated suppression of AR transactivation is dependent on CAMKII and AKT signaling pathways.

We further analyzed the expression of CAMKIIα, CAMKIIβ, CAMKIIγ and CAMKIIδ genes in prostate cancer cell lines (LNCaP, C4-2, DU145, and PC3) by qRT-PCR (Supplemental Figure 3A-C). The prostate cancer cells exhibited increased expression of CAMKIIβ (Supplemental Figure 3A) compared to RWPE1, a normal prostate cell line. CAMKIIα expression was not detected in all prostate cell lines tested. Next, we used qRT-PCR to determined whether suppression of CAMKII is mediated by CAMK2N1. We observed that knockdown of CAMK2N1 in LNCaP and C4-2 cells increased expression of CAMKII, while overexpression of CAMK2N1 in DU145 and PC3 cells decreased expression of CAMKII (Supplemental Figure 3D-F). These findings indicate that CAMK2N1 inhibits the expression of CAMKII (particularly the CAMKIIβ isoform) in prostate cancer cells.

### CAMK2N1 contributes to castration-sensitive cell growth through AR-dependent signaling

**Figure 5:**
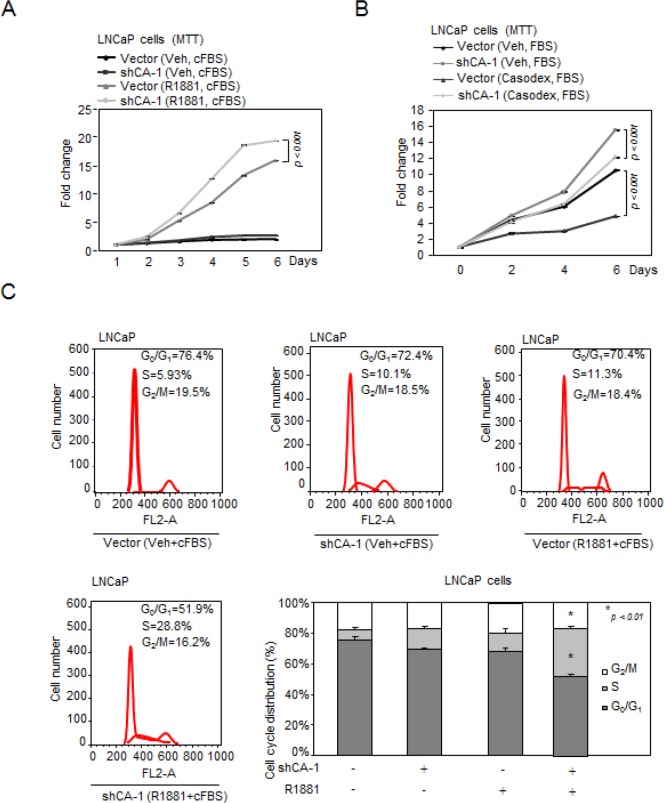
CAMK2N1 contributes to castration-sensitive cell growth through AR-dependent signaling (A) LNCaP cells with stable knockdown of CAMK2N1 were treated with or without R1881. Cells were analyzed for cell proliferation by MTT assay. (B) LNCaP cells with stable knockdown of CAMK2N1 followed by treatment with or without Casodex (10 μM) were analyzed for cell proliferation by MTT assay. (C) LNCaP cells with stable knockdown of CAMK2N1 were treated with or without R1881. Cells were then analyzed for cell cycle by flow cytometry.

To determine the functional significance of CAMK2N1 in regulating the proliferation of AR-positive CSPC cells, we conducted MTT assays and found that knockdown of CAMK2N1 in LNCaP cells using shRNAs increased cell proliferation (Fig. 5A-B, Supplemental Fig. 4A).

AR agonist, R1881, stimulates castration-sensitive cell growth [17]. AR antagonist Casodex, a pharmaceutical drug commonly is used as an anti-androgen therapy to treat recurrent prostate cancer. The growth of castration-sensitive cells is inhibited by Casodex. To further investigate whether CAMK2N1 inhibition of castration-sensitive cell proliferation is dependent on AR, we knocked down CAMK2N1 in LNCaP cells followed by R1881 or Casodex treatment. As shown in Figure. 5A-B, we observed that depletion of CAMK2N1 increased LNCaP cell growth in the presence of R1881, while alleviated inhibition of cell growth to Casodex. Taken together, this data suggests that AR-dependent signaling was involved in CAMK2N1 repression of castration-sensitive cell proliferation.

FACS analysis was performed to further characterize the effects of CAMK2N1 on cell-cycle progression. We knocked down CAMK2N1 in LNCaP cells followed by R1881 treatment. Depletion of CAMK2N1 reduced the proportion of cells in G0/G1 but increased the proportion of cells in S-phase in the presence of R1881, while androgen-deprivation alleviated the effects of knockdown CAMK2N1, suggesting that AR-dependent signaling was involved in CAMK2N1 repression of cell cycle progression (Fig. 5C).

### CAMK2N1 contributes to castration-resistant cell growth through AR-dependent signaling, and re-introduction of CAMK2N1 revert castration-resistance of prostate cancer cells

**Figure 6:**
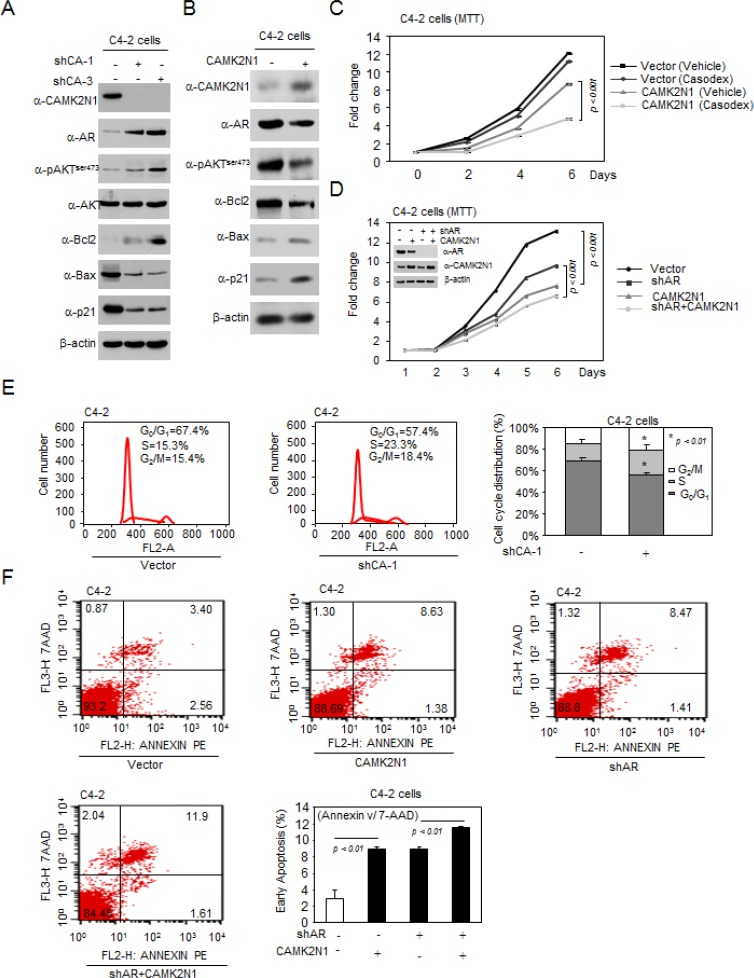
CAMK2N1 contributes to castration-resistant cell growth through AR-dependent signaling, and re-introduction of CAMK2N1 revert castration-resistance of prostate cancer cells (A) Expression levels of pAKT^ser473^, AKT, AR, Bcl-2, BAX, and p21 were determined by Western blot in C4-2 cells with stable CAMK2N1 knockdown. (B) Expression levels of pAKT^ser473^, AR Bcl-2, BAX, and p21 were determined by Western blot in C4-2 cells with stable CAMK2N1 overexpression. (C) C4-2 cells with stable overexpression of CAMK2N1 and AR knockdown. Cells were analyzed for cell proliferation by MTT assay. CAMK2N1 and AR protein levels were determined by Western blot. (D) C4-2 cells with stable overexpression of CAMK2N1 followed by treatment with or without Casodex (10 μM) were analyzed for cell proliferation by MTT. (E) C4-2 cells with stable knockdown of CAMK2N1 were analyzed for cell cycle by flow cytometry. (F) C4-2 cells with stable overexpression of CAMK2N1 and AR knockdown. Cells were analyzed for cell apoptosis by Annexin V staining.

To further validate CAMK2N1-regulated signaling pathways in CRPC cells, a subset of genes that were AR, AKT, pAKTser473, Bcl-2, p21 and Bax were determined by Western blot. Depletion of CAMK2N1 by shRNA in C4-2 cells resulted in increased AR, pAKTser473, and Bcl-2 expression and decreased p21 and Bax expression (Fig. 6A). Conversely, overexpression of CAMK2N1 in C4-2 cells led to an opposite effect on protein expression of these genes (Fig. 6B).

AR has been reported to promote cell proliferation and plays a critical role in the development of CRPC [3, 18]. Knockdown of AR in castration-resistant C4-2 cells reduced cell growth [19]. To further investigate whether CAMK2N1 inhibition of CRPC cell proliferation is dependent on AR, we first knocked down CAMK2N1 in C4-2 cells. Depletion of CAMK2N1 increased C4-2 cell growth and promoted cell cycle progression (Fig. 6E, Supplemental Fig. 4B). Furthermore, we overexpressed CAMK2N1 with simultaneous depletion of AR in C4-2 cells. CAMK2N1 inhibited cell proliferation in the presence of endogenous AR (Fig. 6C), while AR knockdown alleviated the CAMK2N1 repression. This data suggests that AR-dependent signaling was involved in CAMK2N1 repression of cell proliferation.

One of the hallmarks of prostate cancer treatment failure is the development of castration resistance to anti-androgen therapies. Ligand-independent AR activation contributed to the development of castration resistance [18, 20]. It has been shown that AKT phosphorylation and activity stimulated ligand-independent AR activation could contribute to castration resistant growth of prostate cancer cells [21, 22]. In our study, CAMK2N1-mediated suppression of AR activation is dependent on the AKT pathway. To explore whether CAMK2N1 inhibits ligand-independent AR activation and reverts castration resistance, we conducted MTT assays in C4-2 cells treated with Casodex. As a competitive inhibitor of AR, Casodex prevents androgen binding to the AR by blocking its binding sites to the target gene promoter [23]. As a result, castration-resistant cells are refractory to this treatment [24, 25]. As shown in Figure. 6D, we observed that overexpression of CAMK2N1 induced inhibition of growth to Casodex in castration-resistant C4–2 cells, indicating that expression of CAMK2N1 can restore Casodex sensitivity of prostate cancer cells.

Since elevated cell death may also contribute to impaired tumor growth, we determined whether CAMK2N1 induces apoptosis through AR by Annexin V staining assays. We overexpressed CAMK2N1 with simultaneous depletion of AR in C4-2 cells. CAMK2N1 induced cell apoptosis from 3%~8% in the presence of endogenous AR, while AR knockdown alleviated the CAMK2N1 induction from 8%~11% (Fig. 6F). This data suggests that AR-dependent signaling was involved in CAMK2N1 induction of cell apoptosis.

### CAMK2N1 inhibits AR-positive prostate tumor growth *in vivo*

**Figure 7:**
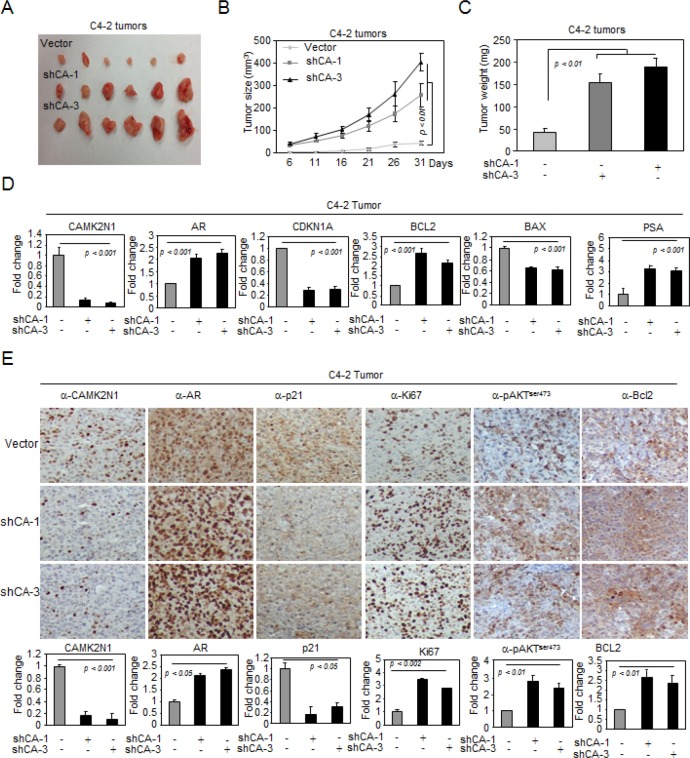
CAMK2N1 inhibits AR-positive prostate tumor growth *in vivo* (A-C) C4-2 tumors with stable CAMK2N1 knockdown were injected into nude mice. Tumor size was measured every 5 days. The data was shown as mean ± SEM for N > 6 separate tumors for each group. (A) Images of tumors dissected from the mice. (B) The tumor size (mm^3^) versus days of post injection. (C) Tumor was weighted after resection at the end of experiment. (D) mRNA levels of AR, PSA, P21, BAX and BCL2 were determined by qRT-PCR in tumors. (E) IHC staining detected the protein expression of CAMK2N1, AR, p21, Ki67, pAKT^ser473^ and BCL2 in C4-2 tumor tissues derived from mice. Data for quantified IHC was shown as mean ± SEM for N = 4 tumors in each group.

To investigate the role of CAMK2N1 in inhibiting AR-positive prostate cancer growth *in vivo*, we established C4-2 cells stably transduced with lentiviral shRNAs targeting CAMK2N1. We implanted these cells subcutaneously into immune-deficient mice and monitored the tumor growth (Fig. 7A-C). Knockdown of CAMK2N1 increased tumor size (Fig. 7A-B) and tumor weight (Fig. 7C).

To determine the expression of CAMK2N1, AR, pAKTser473, PSA, Bax, Bcl-2, p21, and Ki67 in tumor tissues, we conducted qRT-PCR and IHC staining in tumors. As shown in Fig. 7D-E. Tumors with CAM2KN1 knockdown have reduced expression of p21, Bax and increased AR, pAKTser473, PSA, Bcl-2 and Ki67 expression, suggesting that reduction of CAM2KN1 promotes growth in AR positive prostate cancer cells.

## DISSCUSSION

AR plays a critical role in the development of CRPC. In CSPC, AR promotes cell proliferation through regulation of the G_1_/S transition [3, 18]. In CRPC, AR is thought to remain active through a variety of potential mechanisms including AR gene amplification, mutations, abnormal activation, and AR activator overexpression [4, 18]. Upregulation of AR activation in the absence of androgen is one of the mechanisms that lead to abnormal AR activation, which promotes CRPC development [26]. It has been previously shown that AKT serves as positive regulator of AR activity [22]. PI3K/AKT signaling pathways cooperate in prostate cancer progression and its transition to castration resistant disease [27]. Inhibition of PI3K/AKT pathways acts combinatory to inhibit tumor growth, cellular proliferation and migration of prostate cancer [28]. Overexpression of CAMK2N1 suppressed AKT and AR suggests that the loss of CAMK2N1 in advanced prostate cancer could be the primary cause of enhanced kinase signaling, which consequently leads to abnormal activation of AR.

Patients with high levels of AR expression in their tumors are usually associated with decreased recurrence-free survival [29]. Thus, blocking AR expression and activation in clinical practice has become one of the most effective approaches in prostate cancer therapy. Herein, we provided evidence showing in human prostate cancer tissue endogenous CAMK2N1 expression was inversely correlated with AR. Recurrent patients with high AR levels had an improved recurrence-free survival if their tumor also expressed high CAMK2N1 protein levels. In prostate cancer cells, CAMK2N1 and AR signaling form an auto-regulatory negative feedback loop, where CAMK2N1 is down regulated by AR activation in response to androgen, while CAMK2N1 inhibits AR expression and activity through the CAMKII and AKT pathway. We speculate that activation of AR upon androgen binding inhibits CAMK2N1 gene expression in CSPC, and androgen ablation therapy promoted the expression of CAMK2N1, further causing inhibition of AR expression and activation. At the CRPC stage, abnormal activation of AR independent of androgen stimulus leads to the reduced CAMK2N1 expression, which further enhances AR expression and activation.

Androgen ablation therapy is the frontline treatment for patients with advanced prostate cancers including metastatic and castration-resistant diseases. Since the majority of patients eventually develop castration-resistant cancer despite of initial favorable response, identification of molecules and mechanisms involved in castration resistance is critical for developing effective treatment of this deadly disease. We observed that knockdown of CAMK2N1 alleviated inhibition of growth to Casodex in castration-sensitive LNCaP cells and overexpression of CAMK2N1 induces inhibition of growth to Casodex in castration-resistant cells, indicating that re-expression of CAMK2N1 can restore Casodex sensitivity of prostate cancer cells. This study revealed a novel mechanism of CAM2KN1 underlying the development of castration and anti-androgen resistance.

In summary, we identify a biochemical and functional link between CAMK2N1 with reduced expression in advanced prostate cancer and androgen receptor signaling in prostate cancer. Furthermore, loss of CAMK2N1 expression contributes to prostate cancer growth and survival independent of androgen signaling and re-introduction of CAMK2N1 sensitizes castration-resistant prostate cancer cells to anti-androgen therapy. Our results provide a compelling rationale for targeting the functional interaction between CAMK2N1 and AR signaling in clinical development to treat castration-resistant prostate cancer.

## MATERIALS AND METHODS

### Cell culture, Plasmid construction, Reporter genes, Reagents, Expression vectors, and DNA transfection

Human prostate cancer LNCaP cells were obtained from ATCC and maintained in in RPMI 1640 medium (Invitrogen) supplemented with 10% FBS or 10% charcoal/dextran stripped FBS (cFBS). C4-2 was maintained in RPMI 1640 medium (Invitrogen) supplemented with 10% FBS. The CAMK2N1 human cDNA clone was purchased from OriGene Technologies and subcloned into the EcoRI/XhoI site of MSCV-IRES-GFP (Addgen) retroviral vector. pGIPZ lentiviral vector, pGIPZ-shCAMK2N1-1 (shCA-1, 5′-TCAATAACAACCCGCTTGC-3′), pGIPZ-shCAMK2N1-3 (shCA-3, 5′-TAGACACCAGGAGGTGCCT-3′) were purchased from Thermo Scientific. PSA-Luc MMTV-Luc, ARE4-Luc reporter genes were described [30]. AKT inhibitor VIII was purchased from Perkin Elmer (Waltham, MA). LNCaP infected with GIPZ-Vector, shCA-1, shCA-3. C4-2 infected with MSCV-IRES-GFP, MSCV-CAMK2N1-IRES-GFP, pGIPZ-Vector, shCA-1, shCA-3. GFP positive cells were selected by FACS (Fluorescence Activated cell sorter).

### Cell Proliferation Assays

2×10^3^ *stable* cells were seeded in 96-well plate in normal growth medium, and cell growth was measured daily by MTT assays using 3-(4, 5-dimethylthiazol-2-yl)-2, 5-diphenyltetrazolium bromide.

### Cell Cycle and Apoptosis Analysis

Cell cycle parameters were determined by flow cytometry. Stable cells were processed by standard methods using propidium iodide staining of nuclear DNA. Each sample was analyzed by flow cytometry with a FACScan Flow Cytometer (Becton-Dickinson Biosciences, Mansfield, MA) using a 488 nm laser. Histograms were analyzed for cell cycle compartments using ModFit version 2.0 (Verity Software House, Topsham, ME). A minimum of 20,000 events were collected to maximize statistical validity of the compartmental analysis. The PE-Annexin-V Apoptosis Detection Kit (BD Biosciences) was used to detect apoptosis by flow cytometry [31].

### Western Blot

Western blots were performed on DU145, PC3 and LNCaP cells as indicated. Cells were pelleted and lysed in buffer (50 mM HEPES, pH 7.2, 150 mM NaCl, 1 mM EDTA, 1 mM EGTA, 1 mM DTT, 0.1% Tween 20) supplemented with a protease inhibitor cocktail (Roche Diagnostics, Mannheim, Germany). Antibodies used for Western blots were: CAMK2N1 (SC-161427, Santa Cruz) and AR (SC-13062, Santa Cruz).

### Luciferase Assays

Cells were seeded at a density of 1 × 10^5^ cells in a 24-well cell culture plate on the day prior to transfection with Superfect according to the manufacturer's protocol (Qiagen, Valencia, CA). For reporter gene assays, a dose-response was determined in each experiment with 50 and 200 ng of expression vector and promoter reporter plasmids (0.5 μg). Luciferase activity was normalized for transfection efficiency using *β*-galactosidase reporter as an internal control. The fold effect of expression vector was determined with comparison to the value of the empty expression vector cassette and statistical analyses were performed using the t-test [32, 33].

### Chromatin Immunoprecipitation (ChIP) Assays

ChIP assays were performed according to the protocol of the Upstate Biotechnology as described [34]. The primer sequence for PSA gene are PSA-Enhancer: 5′-TGGGACAACTTGCAAACCTG-3′, 5′-CCAGAGTAGGTCTGTTTTCAATCC-3′ [13]. Polyclonal antibody to AR (SC-13062, Santa Cruz) was used for IP and normal IgG was used as a negative control. One tenth of original DNA was used as an input control.

### RNA Isolation and Quantitative Real-time PCR (qRT-PCR) Assays

Total RNA was isolated and reversely transcribed to cDNA using TRIzol reagent (Invitrogen) and iScript cDNA Synthesis Kit (Bio-Rad Laboratories, Hercules, CA), respectively, according to the manufacturer's instructions [35]. qRT-PCR was carried out in Bio-Rad CFX96 Real-Time PCR Detection System with iQ SYBR Green Supermix (Bio-Rad). Relative gene expression was normalized to 18s rRNA and calculated by using the 2-ΔΔCt method.

### Immunohistochemistry (IHC) staining

Immunohistochemical analysis of human prostate cancer was conducted using a polyclonal CAMK2N1 antibody ^8^. Human prostate cancer tissue arrays were purchased from Biomax. FOUR-micrometer sections were prepared from paraffin-embedded C4-2 Tumor tissues derived from nude mice, and tissues were extracted from paraffin. Tumor tissues were stained with primary antibody including Ki67 (RM-9106-S1, Thermo), Bcl-2 (SC-7382, Santa cruz), Bax (SC-7480, Santa cruz), p21 (SC-6246, Santa cruz).

### Nude Mice Study

2 × 10^6^ C4-2 cells knockdown CAMK2N1 were implanted subcutaneously into 4-6-week-old castrated mal nude mice purchased from Beijing HFK Bio-Technology.co., LTD. Tumor growth was measured using a digital caliper every 5 days for 4-5 weeks. Tumor weight was measured when mice were sacrificed on day 32 after cell implantation.

## SUPPLEMENTARY MATERIAL FIGURES


