# Supplementary material for: CAMK2N1 inhibits prostate cancer progression through androgen receptor-dependent signaling
Source: Oncotarget. 2014 Sep 25;5(21):10293–306. doi: 10.18632/oncotarget.2511 (PMC4279373; doi:10.18632/oncotarget.2511)
Supplement: Supplementary file 1 [file oncotarget-05-10293-s001.pdf]

CAMK2N1 inhibits prostate cancer progression through androgen receptor-dependent signaling

Supplementary Material

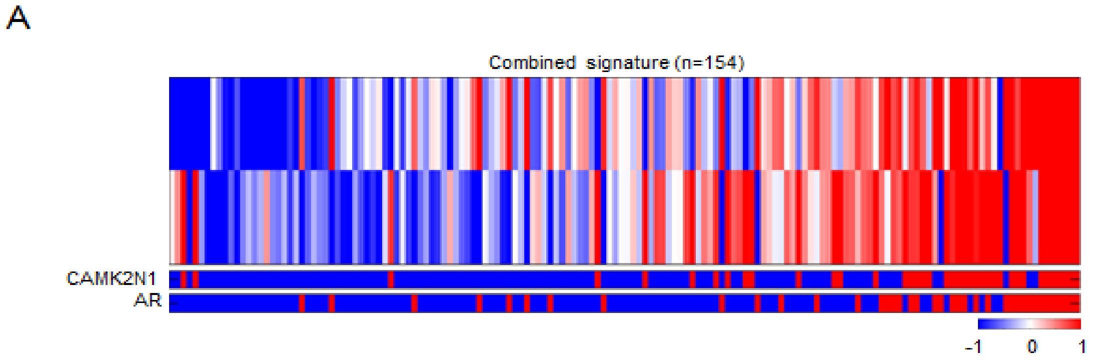

Supplemental Figure 1: Heatmap depicting samples from combined human prostate cancer microarray datasets. (A) Heatmap depicting samples from combined human prostate cancer microarray datasets that were assigned to prostate cancer gene expression subtypes (N = 154).

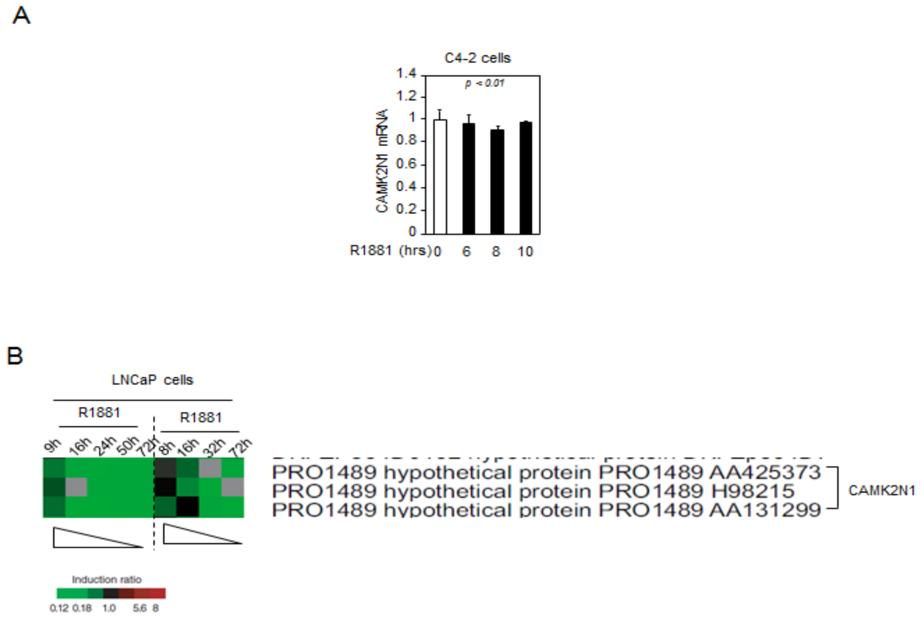

Supplemental Figure 2: Microarray data in the public domains for effects of androgens on CAMK2N1 mRNA expression in LNCaP cell line. (A) C4-2 cells were treated with 10 nM R1881 for 6-10 hrs. CAMK2N1 mRNA levels were determined by qRT-PCR. (B) Hierarchical cluster analysis of CAMK2N1 genes in LNCaP cells treated with R1881. CAMK2N1 mRNA levels were decreased upon androgen treatment in LNCaP cells.

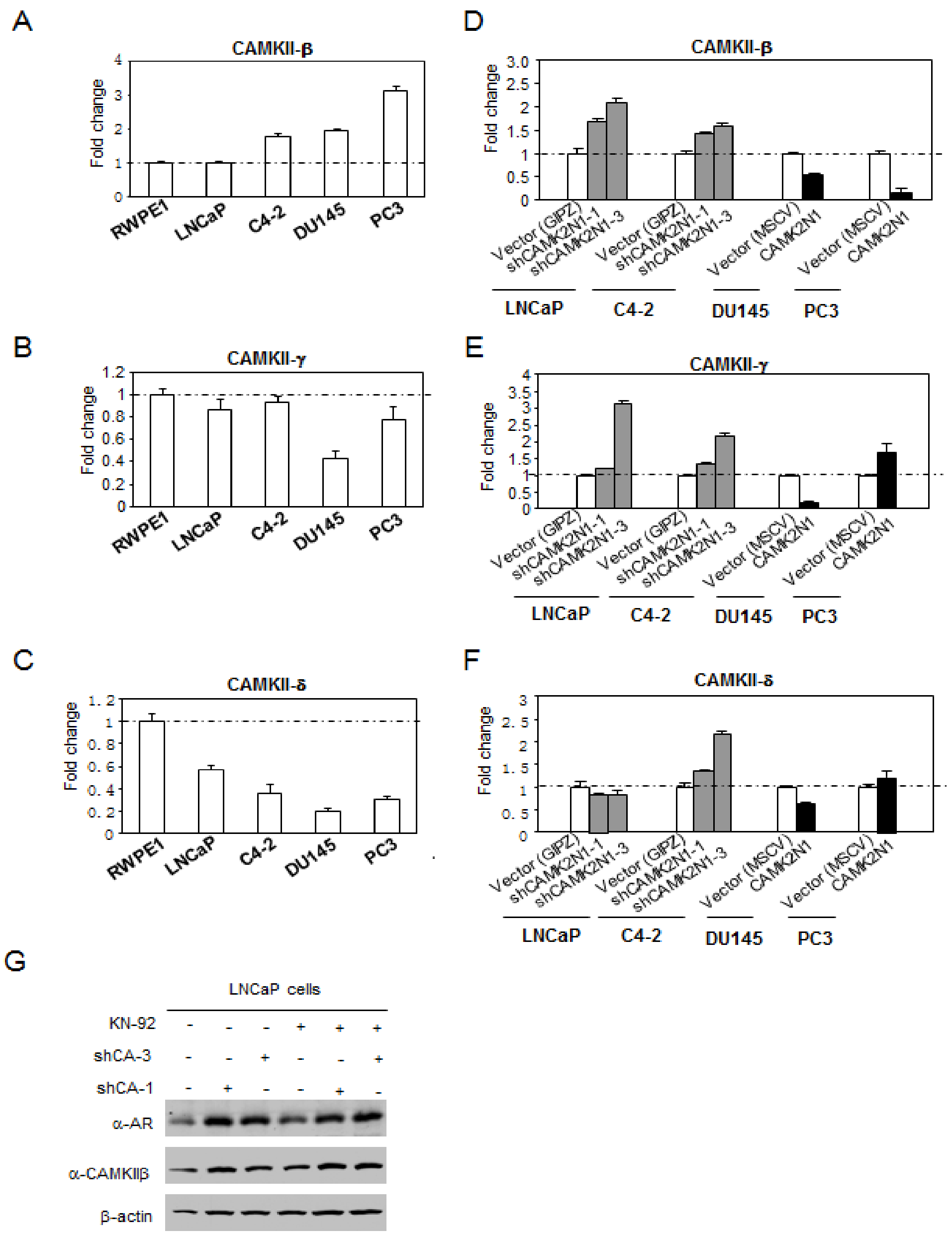

Supplemental Figure 3: CAMKII gene expression in prostate cancer cell lines. (A-C) qRT-PCR analysis of CAMKII mRNA levels in prostate cancer cell lines (RWPE1, LNCaP, C4-2, DU145, and PC3). RWPE1, which is normal prostate cells used as a control, showed lower expression of CAMKIIβ. All of the prostate cancer cells exhibited increased expression of CAMKIIβ. CaMKIIα expression was not detected in any prostate cell lines tested (data not shown). (D-F) LNCaP and C4-2 cells stably knocked down CAMK2N1. DU145 and PC3 cells stably overexpressed CAMK2N1. qRT-PCR analysis of CAMKII mRNA levels in these prostate cancer cell lines. CAMK2N1 significantly inhibited the expression of CAMKII. (G) CAMK2N1 knockdown cells treated with 10 nM R1881 and/or 20μM KN-92 inhibitor. CAMKIIβ and AR protein levels were determined by Western blot.

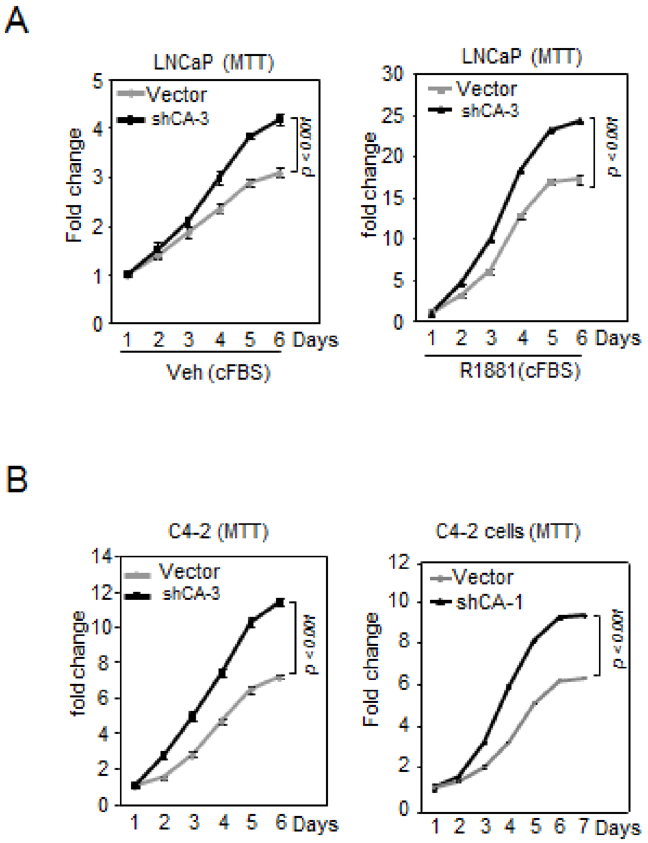

Supplemental Figure 4: CAMK2N1 inhibits cell proliferation in LNCaP and C4-2 cells. (A) LNCaP stably knocked down CAMK2N1 with or without R1881 treatment. Cells were analyzed for cell proliferation by MTT. (B) C4-2 cells with stable knockdown of CAMK2N1. Cells were analyzed for cell proliferation by MTT assay.
